# Laboratory and pilot-farm evaluation of cleaning and disinfection protocols for biofilms in livestock drinking water systems

**DOI:** 10.1016/j.bioflm.2026.100397

**Published:** 2026-09-11

**Authors:** Ulric Van Rossum, Marc Heyndrickx, Niels Demaître, Geertrui Rasschaert, Nico Boon, An Cools, Koen De Reu

**Affiliations:** a Technology and Food Unit, Flanders Research Institute for Agriculture, Fisheries and Food, Merelbeke-Melle, 9090, Belgium; b Inagro, Rumbeke-Beitem, 8800, Belgium; c Evap Proefbedrijf Pluimveehouderij, Geel, 2440, Belgium; d Faculty of Bioscience Engineering, Centre for Microbial Ecology and Technology, Ghent University, Ghent, 9000, Belgium; e Faculty of Veterinary Medicine, Ghent University, Ghent, 9000, Belgium

**Keywords:** Biofilms, Disinfection, Water quality, Livestock, CDC biofilm reactor

## Abstract

Persistent biofilms harboring pathogens can compromise drinking water quality in livestock housing, leading to animal health issues and reduced production. Microbial water quality can be maintained through various treatments and/or by cleaning and disinfecting drinking water systems to remove dirt and biofilms. However, despite routine monitoring of drinking water quality, biofilm persistence after treatment is often overlooked in practice. This study evaluated commonly used (cleaning and) disinfection protocols (peroxides, peracetic acid, chlorine dioxide, formic acid, chlorine, and enzymatic) for drinking water systems, both *in vitro* and *in situ*. Four selected three- or four-species communities were grown on polycarbonate and stainless-steel coupons in a stirred Centers for Disease Control (CDC) biofilm reactor. The *in situ* study was conducted on a pilot broiler farm during four consecutive six-week production cycles, with both water and biofilm samples collected during each cycle. Our *in vitro* study showed that the disinfectants reduced multispecies viable biofilm cells, but the effects varied with microbial composition. The *in situ* results showed little effect of the cleaning and disinfection protocols. Consequently, targeted intervention strategies, supported by regular monitoring of water quality and biofilm presence, are essential for making treatment decisions to minimize the risk of water contamination.

## 1 Introduction

Biofilms are structured, three-dimensional microbial communities that develop on biotic or abiotic surfaces or as non-attached aggregates [1]. Within these communities, microorganisms are encapsulated in a self-produced extracellular polymeric substance (EPS) matrix that promotes adhesion, rapid cell-to-cell interactions, enzymatic activities and functions as a protective barrier, protecting against environmental stress, retaining nutrient sources, and facilitating genetic exchange [2]. Unwanted biofilms occur in many industrial and domestic sectors, such as healthcare, food production, agriculture, and wastewater systems, leading to major economic losses [3]. Furthermore, biofilms in drinking water systems (DWS) can act as environmental reservoirs for pathogens, increasing the risk of drinking water contamination and posing a potential risk to human and animal health [4,5]. Following the European Animal Health Law, farmers must ensure animal health and prevent diseases by, for instance, procuring safe water for the animals [6]. This is especially important for piglets and for broilers, as these young animals are more susceptible to pathogens [7,8]. Livestock farmers can use water from different sources, such as wells, rainwater, surface water, drainage, or tap water. These may vary in quality and therefore require measures to improve microbiological quality. There is no standard solution, but depending on the water source, typical (combined) measures are water filtration, UV disinfection, acidification, and continuously adding a low dosage of disinfectants. Additional measures to ensure water quality include cleaning and disinfecting (C&D) of DWS, which may be performed separately or together, combining mechanical actions such as flushing with the application of disinfectants (e.g., oxidative or chlorine-based agents) to remove biofilms and other organic and inorganic contaminants. [9,10]. Farmers collect water samples at specific times based on animal specifications and primarily analyze them in laboratories for culturable microorganisms, following ISO standards. [[11], [12], [13]]. The presence of biofilms in DWS is, in contrast, not routinely tested, although it may still occur and pose potential risks [4,5,[14], [15], [16], [17], [18]].

In our previous *in vitro* research, we reported increased biofilm biomass in microtiter plate experiments with several four-species communities compared to the cumulative biomass of the involved species grown in single culture [19]. These multispecies biofilms contained bacterial species isolated from biofilm samples collected from the inner surfaces of DWS lines in broiler houses and pig nursery units, with a dominance of *Staphylococcus* and *Pseudomonas* species (e.g., *Staphylococcus paraoxydans*, *Pseudomonas aeruginosa* and *Pseudomonas fluorescens*) [5]. Furthermore, the *in vitro* multispecies combinations included two common species, *Citrobacter freundii* and *Staphylococcus nepalensis*, because their presence in such combinations was associated with increased biofilm biomass production through synergistic interactions [19]. Both species are also recognized as opportunistic pathogens [20,21]. The transferability of commercial C&D efficacy from simplified laboratory tests to multispecies biofilms in operating livestock water lines remains poorly established. Therefore, this study aimed to (i) characterize the development of selected multispecies biofilms on polycarbonate and stainless-steel coupons; (ii) compare five disinfection protocols using residual culturable counts in a CDC reactor mode; and (iii) evaluate six protocols by monitoring water and biofilm-associated culturable counts in a pilot broiler facility over four production cycles. The findings of this study provide important insights into the effectiveness of C&D protocols on multispecies biofilms in DWS.

## 2 Materials and methods

### 2.1 *In vitro* monitoring of disinfection practices of drinking water systems with a model multispecies biofilm

#### 2.1.1 Model multispecies biofilms

Four multispecies combinations were selected as *in vitro* biofilm models from DWS at livestock barns, with 8 bacterial isolates recovered from biofilm samples in DWS of pig nursery units and 4 from DWS of broiler houses. Based on previous research, combinations with the highest synergistic interactions were selected (Table 1; [19]). All isolates were identified as predominant species in the microbiota from DWS biofilm samples [5].

**Table 1:** Four multispecies combinations with isolates originating from drinking water system biofilm samples at pig nursery units and broiler houses.

Combination	Species	Tag	Origin
1.	*Staphylococcus nepalensis*	MB6805	Pig nursery unit
	*Pseudomonas aeruginosa*	MB6806	
	*Microbacterium paraoxydans*	MB6807	
2.	*Staphylococcus nepalensis*	MB6805	Pig nursery unit
	*Pseudomonas aeruginosa*	MB6806	
	*Microbacterium esteraromaticum*	MB6808	
	*Psychrobacter pulmonis*	MB6809	
3.	*Staphylococcus nepalensis*	MB6805	Pig nursery unit
	*Aerococcus viridans*	MB6810	
	*Microbacterium paraoxydans*	MB6811	
	*Citrobacter freundii*	MB6812	
4.	*Acidovorax delafieldii*	MB6813	Broiler house
	*Brevundimonas diminuta*	MB6814	
	*Pseudoacidovorax intermedius*	MB6815	
	*Pseudomonas aeruginosa*	MB6816	

#### 2.1.2 Kinetic analysis of biofilm mass development in the CDC Biofilm Reactor®

The CBR 90 Standard CDC Biofilm Reactor® (Biosurface Technologies Corporation, Bozeman, MT, USA) was prepared and sterilized according to the manufacturer's instructions. All isolates were first grown overnight in 50% Brain Heart Infusion broth (BHI; Oxoid, Hants, United Kingdom; CM1135) and then diluted in 400 ml 50% BHI in the reactor to a final OD_600_ of 0.01 (∼10^6^ cells/ml), ensuring equal representation of each species in the multispecies (3 or 4 species) combination. Biofilms were formed on 304L stainless steel and polycarbonate coupons (Biosurface Technologies Corporation; RD128-304 and RD128-PC), each with an area of 1.26 cm^2^, for 48 h at 25°C under continuous stirring at 60 rpm. At seven time points (0, 4, 7, 24, 28, 31 and 48h), 1 mL of the inoculated solution was sampled, and a rod holding three coupons was removed. Biofilm biomass from each coupon was recovered in 1 ml of Ringer's solution (Oxoid, BR0052G) by scraping with a sterile wooden stick, followed by sonication at 40 kHz (Branson 2510, Danbury, CT, USA) and homogenization at 3200 rpm (Biosan MPS-1, Riga, Latvia), both for 10 min [22,23]. Both the solution with the planktonic cells and the suspension obtained from the biofilms were serially diluted in 10 ml Buffered Peptone Water (BPW; BioTrading, Berlin Germany, K168B009AA) and spiral-plated on BHI agar (Oxoid; CM1136) and on selective media to allow enumeration of each species individually (Table 2), except for *B. diminuta*, *A. delafieldii*, and *P. intermedius*, for which selective media could not be identified in the literature. The plates were then incubated at 25°C for 72h, except for *C. freundii* and *A. viridans,* which were incubated at 37°C for 72h [24,25]. The four combinations were tested in triplicate, yielding five coupons per material type. The lower limit of quantification (LLOQ) was 1.3 log CFU/cm^2^ for all culture media.

**Table 2:** Selective media used to count the species individually in the multispecies biofilms.

Species	Culture media	Supplement(s)	Reference
*Citrobacter freundii*	RAPID’*E.coli* 2 Agar (Bio-Rad Laboratories, Hercules, US, 17005373)	n.a.	[24]
*Staphylococcus nepalensis*	Plate Count Agar (ThermoFisher, CM0463B)	10% NaCl (w/v)	[26]
*Microbacterium* spp.	Nutrient Agar (ThermoFisher, CM0309B)	0.005% colistin and 0.0001% kanamycin (w/v)	[27]
*Psychrobacter pulmonis*	Tryptone Soy Agar (ThermoFisher, CM0131B)	6.5% NaCl and 0.1% crystal violet (w/v)	[28]
*Pseudomonas aeruginosa*	Pseudomonas Agar Base (ThermoFisher, CM0559B)	C-F-C supplement (ThermoFisher, SR0103E)	n.a.
*Aerococcus viridans*	Slanetz & Bartley (ThermoFisher, CM0377B)	n.a.	[25]

n.a. = not applicable.

#### 2.1.3 Monitoring of disinfection protocols in the CDC biofilm Reactor®

In the second experiment, biofilm mass was grown on stainless steel and polycarbonate coupons in the CDC Biofilm Reactor® for 48 h at 25°C in 50% BHI, as described previously. After incubation, each rod with three coupons was removed, rinsed with sterile water by immersion, and transferred to 50 ml Falcon tubes containing 30 ml of each of the five disinfection products (A to E) listed in Table 3. They were incubated at 25°C using the suppliers' recommended concentrations and contact times as used in practice. Coupons were then rinsed again by immersion in sterile water, after which the biofilm masses were recovered as previously described.

**Table 3:** Disinfection products for drinking water systems analyzed in this study. Their active compound, product concentration, the final active compound concentration added to water, and the contact time at which the water was held in the DWS prior to flushing.

Product	Active compound(s)	Product concentration (v/v)	Active compound (s) concentration (v/v)	Contact time (h)
A.	Hydrogen peroxide (H_2_O_2_)	2%	0.99%	6h
B.	H_2_O_2_ and peracetic acid (PAA)	2%	0.42% and 0.11%	4h
C.	H_2_O_2_ and formic acid (FA)	3%	1.35% and 0.0026%	12h
D.	ClO_2_	1.5%	0.0053%	4h
E.	Enzymes followed by PAA	2% for both	2.00% and 0.04%	6h and 2h
F.	Cl_2_ (gas)	n.a.	0.017%	Gas recirculation

n.a. = not applicable.

The resulting suspensions were serially diluted and plated onto BHI agar and selective culture media to enumerate the specific bacterial species (Table 2). A total of nine coupons for each material type and each multispecies combination were collected across three independent experiments.

### *2.2* *In situ* monitoring of cleaning and disinfection practices of drinking water systems in a broiler house

**Fig. 1:**
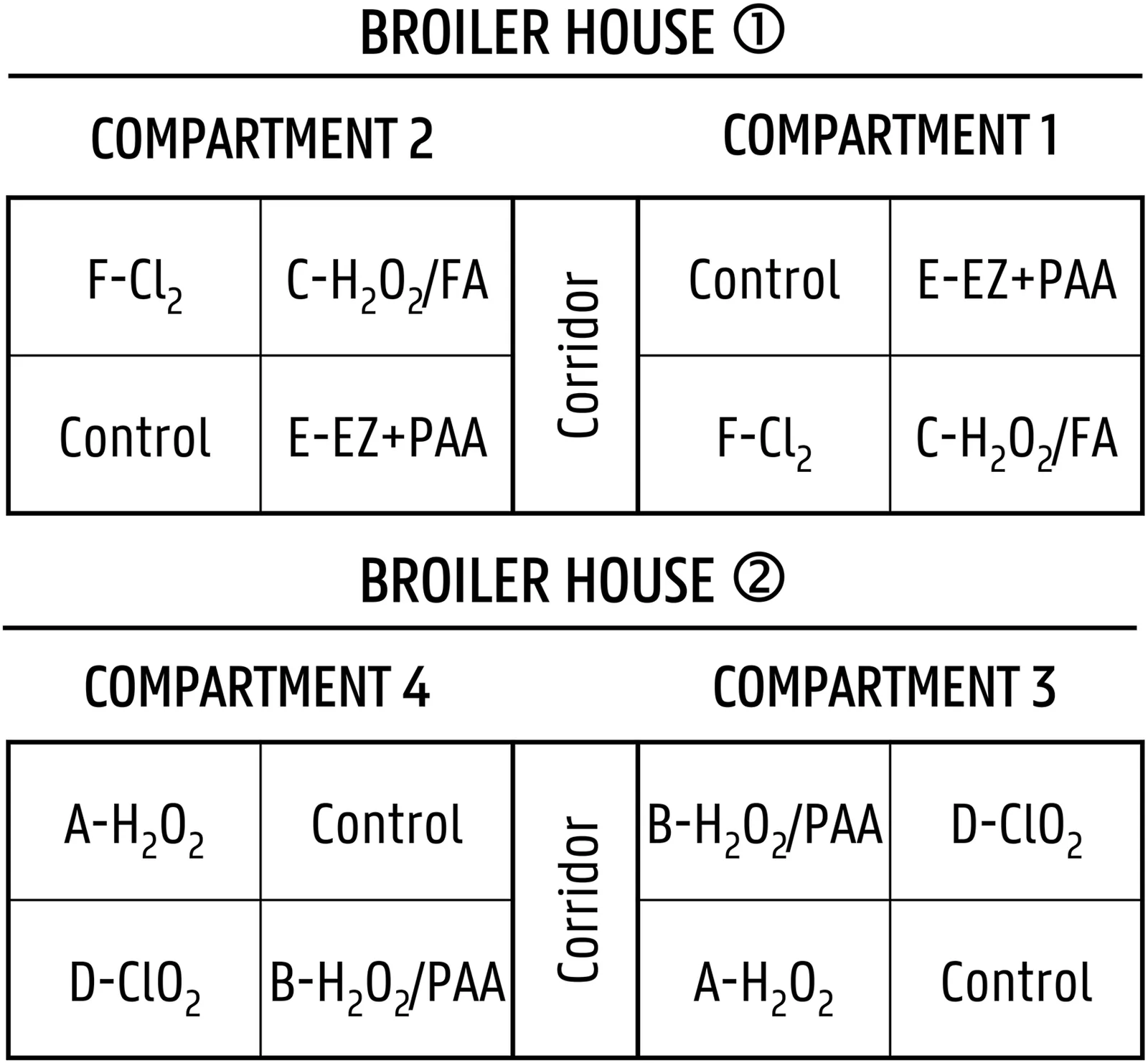
Pilot broiler farm setup for testing cleaning and disinfection protocols. Two broiler houses were each divided into two compartments (1 and 2; 3 and 4), and each stable was further divided into four pens. The three drinking water lines in each pen were treated with disinfection products (A, B, C, D, E, and F), and one pen in each stable served as a control.

The C&D protocols for DWS listed in Table 2 were also evaluated on a pilot broiler farm during four consecutive animal production rounds, each lasting six weeks, with downtime of no more than one week, from January until July 2025. In addition to the disinfection products used in the *in vitro* tests, a product containing free chlorine gas (product F) was tested. The C&D protocols were applied during downtime, first flushing the drinking water pipelines to clean them, then adding the products at the recommended concentrations to the water to be filled in the DWS and held for the recommended time. The pilot farm is divided into two poultry houses; each house contains two compartments, each with four pens (1, 2, 3, and 4) with three drinking water lines, each with 25-30 drinking nipples. Three of the four pens were treated with a different product. The remaining pen was kept as control where only flushing was applied (Fig. 1). In stables 1 and 2 only tap water was used, while in stables 3 and 4 tap water and rainwater were used so that product A, B and D were combined with both water types (Fig. 1). Both types of water were treated during all the production cycles with a low concentration of a product containing hydrogen peroxide (0.2% v/v) and peracetic acid (0.05% v/v). During the four production rounds, biofilm samples from the inside of the DWS and drinking water samples were collected at different times: during the downtime, within the first four days after C&D of the broiler house and DWS, and later during production at broiler ages of two and five weeks. The samples were collected as described before [5]. Briefly, biofilm samples were collected using cotton swabs moistened with Dey-Engley Neutralizing Buffer (Sigma-Aldrich, Diegem, Belgium; D3435). Samples were collected from approximately 20 cm^2^ of the inner surfaces of the drinking lines at the drinking nipples after removing the water and nipples. Each sample was taken from a different drinking nipple to ensure that undisturbed biofilm was collected. For each water type, 2 to 5 swabs were collected. Inflow water samples (1L) were collected in the building corridor before any product dosing (Fig. 1), and outflow water samples were collected at the end of the drinking lines in each pen. Both sample types were analyzed for viable cell counts at 22°C and 36°C on BHI agar as described before [5].

### 2.3 Statistical analysis

All statistical analyses were conducted using GraphPad Prism (v10.6). Differences in viable biofilm cells between PC and SS coupons, and between the different combinations at each time point were assessed using a two-way repeated-measures analysis of variance (ANOVA) with Geisser–Greenhouse correction. Šídák's multiple-comparisons test was used for pairwise comparisons at each time point (*p* < 0.05). For the *in vitro* experiments, viable biofilm cells on treated coupons were compared using a two-way ANOVA with multiple comparisons, followed by Tukey's post hoc test to identify significant differences (*p* < 0.05). The *in situ* results were analyzed using a two-way ANOVA followed by Dunnett's post hoc test to identify significant differences with the control (*p* < 0.05).

## 3 Results

### 3.1 Kinetic analysis of biofilm development in four multispecies combinations

Four multispecies combinations were cultured in 50% BHI in a biofilm reactor on two materials (PC and SS) at 25°C for 48h. Afterward, biofilm biomass was collected, homogenized, and plated on selective and non-selective culture media at seven time points (Table A1). Immediately after inoculation, the average attached cell concentration ranged from 2.9 to 3.8 log CFU/cm^2^ on SS and from 3.6 to 4.8 log CFU/cm^2^ on PC across the four combinations. A statistically significant increase in viable biofilm cells was observed across all combinations over time (*p*-values in Table A2). After 24h, values ranged from 5.1 to 6.1 log CFU/cm^2^ on SS and from 6.1 to 6.9 log CFU/cm^2^ on PC, and further increased to 5.7 to 7.2 log CFU/cm^2^ on SS and 5.7 to 7.8 log CFU/cm^2^ on PC after 48h. Significant differences in viable biofilm cell counts between PC and SS were observed for combinations 1, 2, and 3 (Table A2), with PC showing higher cell counts over the sampled period. In contrast, no significant difference was observed for combination 4 (*p* = 0.2196), in which both materials exhibited similar average bacterial biofilm counts of 5.7 log CFU/cm^2^ after 48h. However, the effect did not significantly interact with the time of sampling. When comparing SS and PC at individual time points with Šídák correction, only the 28h time point in combination 1 showed a statistically significant difference (*p* = 0.0015). Furthermore, comparing total viable biofilm cells across combinations at each time point revealed variable differences among groups *(p-*values in Table A3), with significant differences observed on PC coupons after 48h in combinations 1-3 versus combination 4.

**Fig. 2:**
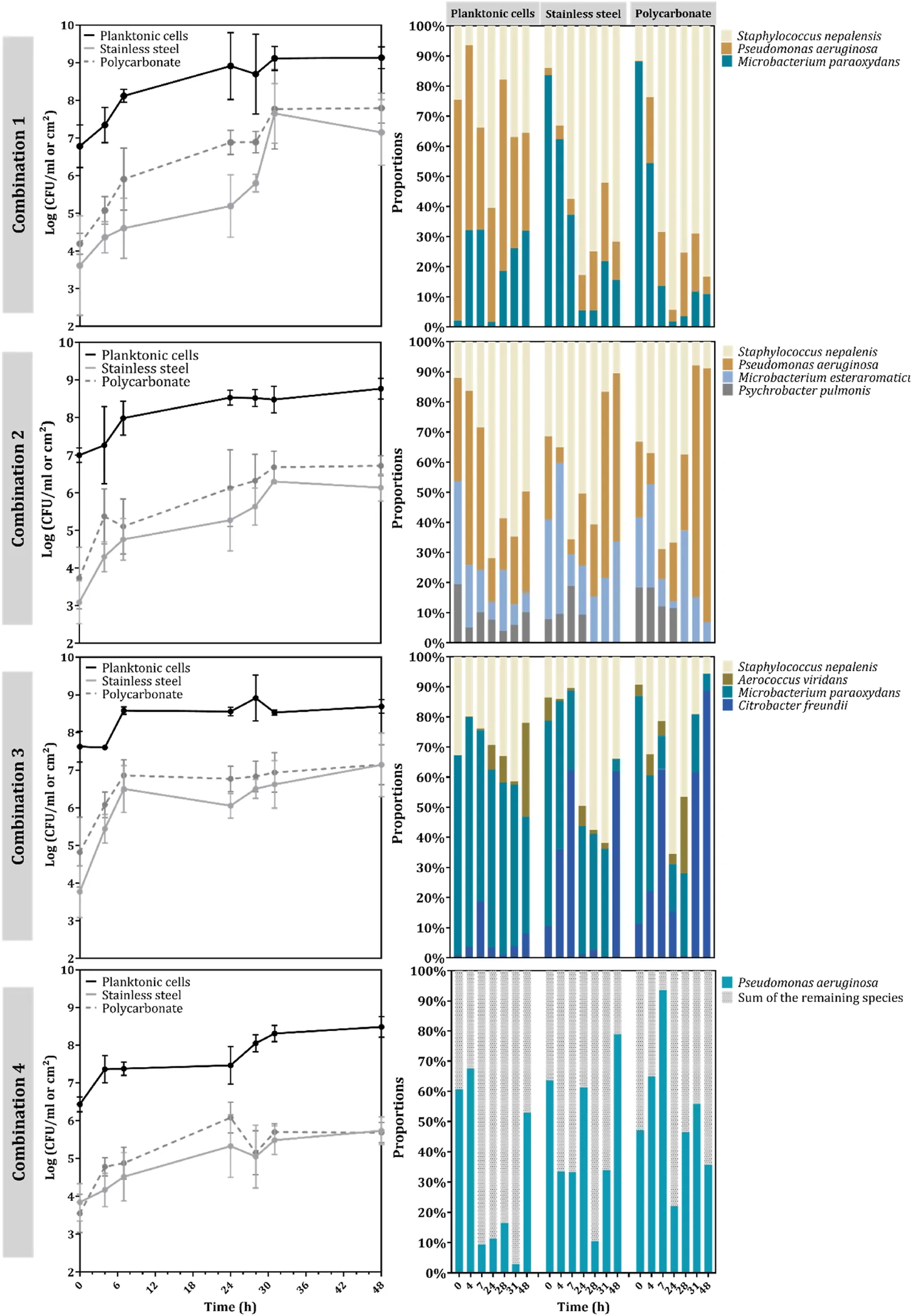
Growth kinetics of biofilm mass of four multispecies biofilms (n = 5) and the respective planktonic cell counts (n = 3) together with the proportions of the species from each combination. The proportions were calculated by dividing the counts for each individual species by the total counts from all selective plates. The sum of the remaining species in combination 4 includes *A. delafieldii*, *B. diminuta* and *P. intermedius.* The LLOQ is 1.3 log CFU/cm^2^ or ml.

Analysis of the biofilm species proportions indicated that *M. paraoxydans* was dominant at 0h and 4h in combinations 1 and 3, whereas by the end, dominance shifted to *S. nepalensis* in combination 1 and to *P. aeruginosa* in combination 2. (Fig. 2). Moreover, after 24h in combination 2, *P. pulmonis* was not countable on any of the coupons and in combination 3, *A. viridans* was not countable after 28h, although they were still present as planktonic cells in the bulk fluid. These proportions differed from those observed in the planktonic cells. In combination 1, *P. aeruginosa* occurred at higher proportions, with a more even distribution among the three species across all sampling time points. In combination 2, *S. nepalensis* remained the dominant planktonic species after 7h, while *C. freundii* was dominant after 48h in combination 3.

### 3.2 *In vitro* disinfection of multispecies biofilms from biofilm reactors

**Fig. 3:**
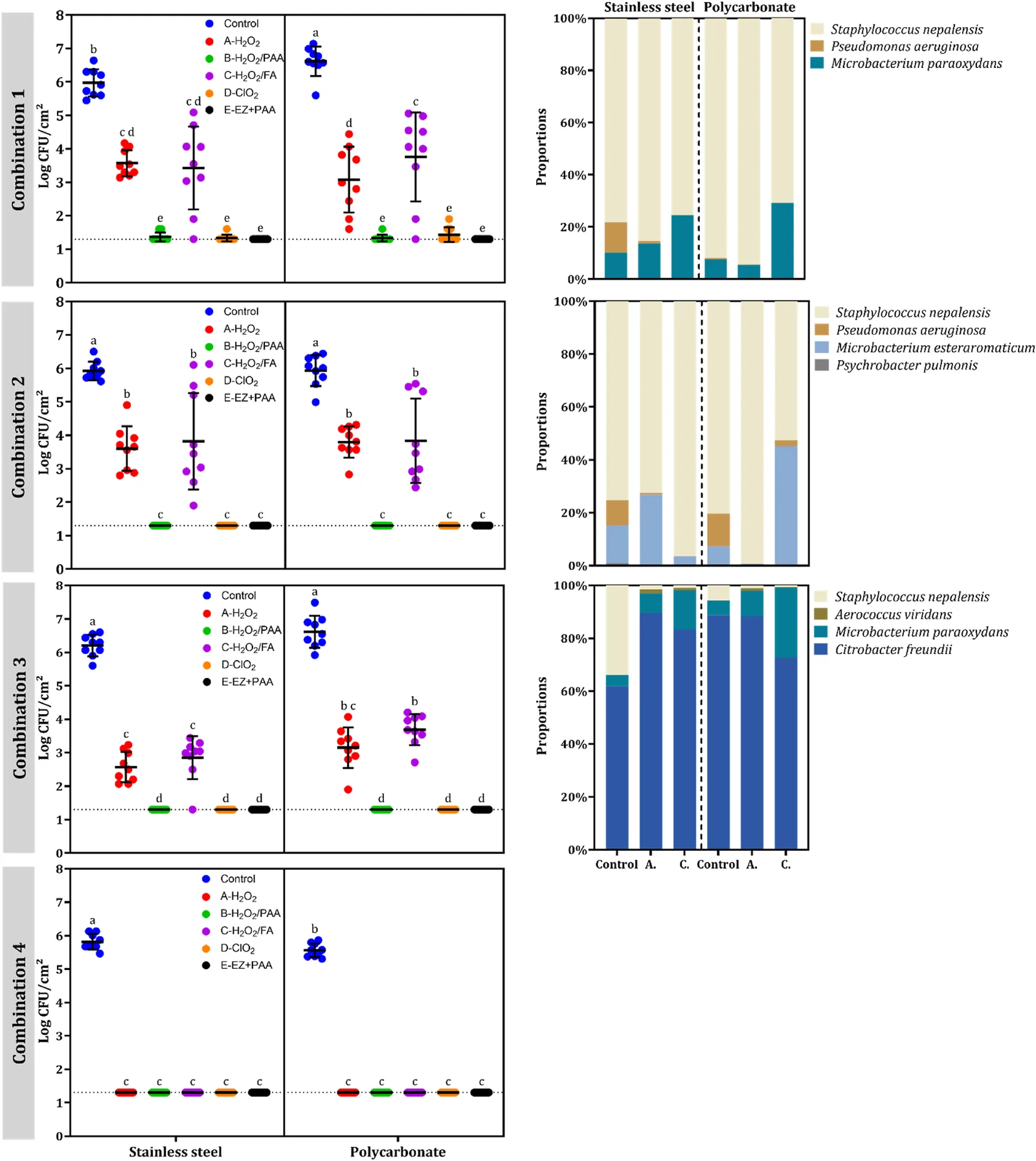
Cleaning and disinfection analyses on biofilm mass produced in the CDC Biofilm Bioreactor® with four multispecies combinations of isolates from water lines in broiler houses and pig nursery units. Cell enumerations after cleaning and disinfection and proportions of the different species within each combination are shown. The dotted line shows the lower limit of quantification (1.3 log CFU/cm^2^). Five different protocols were applied during three independent experiments for each coupon type (n = 9) and significant differences were based on the post-hoc Tukey's test (*p* < 0.05). PAA = Peracetic Acid, FA = Formic Acid and EZ = Enzyme.

After 48h of biofilm growth in the reactor at 25°C, five disinfection protocols for DWS were applied to the coupons, and the remaining biofilm was then collected. For combinations 1, 2, and 3, similar trends were observed (Fig. 3), with treated biofilms showing significantly lower cell counts (*p* < 0.05) than untreated controls across all five products, although differences among products were evident. No differences were observed between the two material types. Products B, D, and E achieved at least a 5-log reduction, whereas products A and C resulted in reductions of 2 to 4 logs. For combination 4, no viable cells were countable following treatment with any of the products. Analysis of the proportions of the remaining countable cells after disinfection using products A and C indicated that *S. nepalensis* remained the dominant species in combinations 1 and 2, while *C. freundii* predominated in combination 3. Additionally, across all three combinations treated with product C, the two *Microbacterium* species emerged as the second-most dominant genus.

### 3.3 On-farm monitoring of cleaning and disinfection practices of drinking water systems in a broiler house

**Fig. 4:**
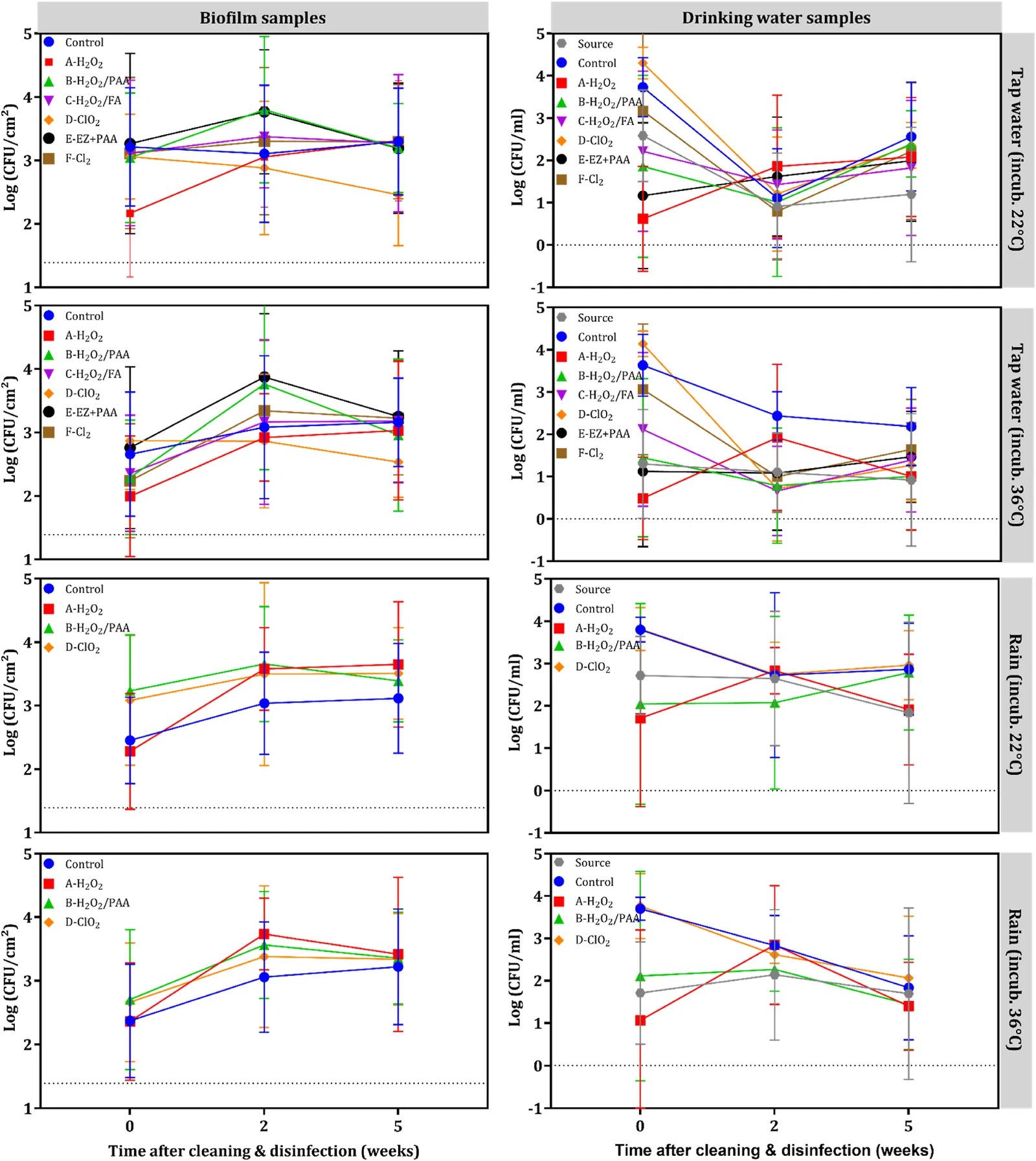
Bacterial enumerations of biofilm swabs and drinking water samples of drinking water systems after cleaning by flushing and disinfection with six different products. The samples were taken after 0 (n_swabs_ = 200 and n_water_ = 75), 2 (n_swabs_ = 128 and n_water_ = 58) and 5 (n_swabs_ = 144 and n_water_ = 76) weeks after cleaning and disinfection during four production rounds each lasting six weeks. The dotted line shows the lower limit of quantification. The specific number of samples are listed in Table A1. PAA = Peracetic Acid, FA = Formic Acid and EZ = Enzyme.

Across the four production rounds, Total Aerobic Counts (TAC) at 22°C and 36°C in biofilm samples from DWS were compared between control and C&D DWS (Fig. 4). Viable biofilm cells recovered from tap water after 22°C incubation were significantly lower for product A compared to the control just after C&D (or time point 0) and for product D after 5 weeks for viable biofilms cells recovered after 36°C incubation (*p* = 0.0147 and *p* = 0.0431, respectively). For DWS containing rainwater, product B resulted in significantly higher counts after 22°C incubation after C&D compared to the control (*p* = 0.0392). Analysis of TAC of tap water samples showed that, following C&D product A resulted in significantly lower counts at both incubation temperatures 22°C and 36°C (*p* = 0.0447 and *p* = 0.0157), as did product E (*p* = 0.0192 and *p* = 0.0257), while after two weeks of production, products C and F at 36°C also demonstrated significantly reduced counts (*p* = 0.0360 and *p* = 0.0339) compared to the control. Furthermore, comparison of influent (source) water samples and control samples revealed significantly higher TAC in the controls at 36°C both after C&D and after two weeks (*p* = 0.0116 and *p* = 0.0432). No significant differences were observed between any of the C&D products and the controls in DWS with rainwater at any sampling moment.

## 4 Discussion

### 4.1 *In vitro* monitoring of disinfection protocols for livestock drinking water systems

There are currently no standard laboratory tests for biocidal efficacy against biofilm [29,30]. Validation of standard practice C&D protocols are mostly lab-verified with planktonic cells or on single-species biofilms ISO norms to test water quality and disinfectant efficacy are focused on planktonic cells [11,[31], [32], [33]]. However, these tests do not account for persistent multispecies biofilms, which can still compromise microbial drinking water quality, leading to higher bacterial loads, increased infection risks, increased antibiotic use, and, in the livestock industry, decreased animal welfare and production [4,5,[14], [15], [16], [17], [18]]. By evaluating standard C&D protocols against biofilms composed of ecologically relevant species, a more realistic assessment of their efficiency in a simulated field context can be achieved. The use of multispecies biofilms underscores the relevance of this approach, as environmental biofilms comprise complex microbial communities in which interspecies interactions strongly influence biofilm structure, development, and stress responses [22,[34], [35], [36], [37]]. In this study, four multispecies combinations (three- or four-species), consisting of isolates obtained from biofilm swabs collected from DWS surfaces [5], were selected as model organisms. Biofilms were cultivated in a static CDC Biofilm Bioreactor® on two representative water-line materials: polymer (PC) and metal (SS) coupons. Compared with commonly used microtiter plate systems, the reactor provides a more representative model by enabling simultaneous evaluation of multiple coupons and allowing controlled mixing to better mimic the shear-stress conditions encountered in DWS systems [23,38]. The selected combinations were based on their positive co-interactions (e.g., synergism, cooperation, and commensalism) and were therefore considered a worst-case scenario for evaluating disinfection protocols [19,39]. When co-cultured, these combinations produced more biofilm biomass than the sum of their respective monocultures, confirming their synergistic behavior [19]. Kinetic analysis over 48h showed that adhesion occurred immediately after inoculation (2.9–4.8 log CFU/cm^2^), followed by a gradual increase in cell concentrations up to 5.7–7.8 log CFU/cm^2^ by the end of the incubation period. For three of the four combinations [[1], [2], [3]], all of which contained *S. nepalensis*, higher cell concentrations were consistently observed on PC than on SS throughout most of the incubation period. This difference was most pronounced during the initial stages of biofilm formation, while by the end of the experiment, cell concentrations on both materials became similar. Polymers, such as PC, are typically more hydrophobic, with lower surface free energy, and therefore promote initial adhesion under water conditions [40,41]. That said, there are many conflicting findings regarding the type of material and adhesion. For instance, the multispecies viable biofilm cells of *Lactobacillus plantarum*, *Leuconostoc pseudomesenteroides*, *Lactobacillus rhamnosus*, and *Pseudomonas fluorescens* were similar across SS, polystyrene, and glass coupons [42]. In single-culture biofilms, higher cell adhesion was observed for *P. aeruginosa* and *Staphylococcus aureus* on SS compared to PC [43,44]. Such contradictory findings have led researchers to hypothesize that adhesion is influenced by a multitude of interacting factors, including the flow and properties of the liquid (e.g., nutrient availability, pH, and temperature) as well as the physical and chemical characteristics of the surface, such as charge, wettability, and roughness [22,41,45].

In addition, bacterial species composition plays a major role in biofilm dynamics and development [39,45]. The resulting viable biofilm cells can be combination-dependent, with average cell counts after 48 h ranging from 6.7 to 7.8 log CFU/cm^2^ for combinations 1–3, compared with only 5.7 log CFU/cm^2^ for combination 4. *M. paraoxydans* and, to a lesser extent, *M. esteraromaticum* appeared to act as early surface colonizers during the first 7h, whereas in later stages they were outcompeted by *S. nepalensis*, *C. freundii*, or *P. aeruginosa*. Early colonizers are known to influence biofilm properties, attachment and growth of secondary colonizers, either facilitating or inhibiting their development [46]. Similar behavior has been reported for the keystone species *Microbacterium lacticum* from the dairy industry, which dominated multispecies biofilms during the first 12 h and promoted the growth of other species before decreasing in abundance due to competition with other bacteria while remaining in the lower biofilm layers [34,39]. The early dominance of *Microbacterium* species on surfaces may be due to their strong adhesion capacity and tolerance to low-oxygen and nutrient-limited conditions [39]. When other species establish themselves, competitive interactions may displace *Microbacterium* to deeper biofilm layers where nutrient availability and metabolic activity are reduced. However, the spatial organization of the biofilms in the present study remains to be confirmed. Furthermore, comparison of biofilm and planktonic cell counts revealed differences in species proportions, with planktonic cells showing a more even distribution among species.

The biofilm-containing coupons were treated with five disinfection protocols using standard applied disinfectants, H_2_O_2_, peracetic acid (PAA), formic acid (FA), and ClO_2_. Similar trends were observed for multispecies combinations 1 to 3, where products B (H_2_O_2_ + PAA), D (ClO_2_) and E (enzymes + PAA) reduced viable biofilm cells to the LLOQ after disinfection, while products A (H_2_O_2_) and C (H_2_O_2_ + FA) achieved a 2 to 4-log reduction. In contrast, the poultry combination 4 was sensitive to all treatments, with no countable cells remaining after disinfection. However, as total biofilm biomass and the susceptibility of individual strains to the biocides were not assessed, the underlying factors contributing to this difference cannot be determined. Nevertheless, these results demonstrate that disinfection efficacy can vary substantially depending on the biofilm community composition, which may be an important consideration for disinfection of DWS. Products A and C both relied on H_2_O_2_, with product C containing a higher H_2_O_2_ concentration with FA, whereas product B combined a lower peroxide dose with PAA, which appeared more effective. The limited penetration and stability of H_2_O_2_ within the biofilm EPS, together with the addition of stabilizers in the product formulation, could explain these results [[47], [48], [49], [50]]. Although combination 4 consisted solely of Gram-negative Proteobacteria, while the other combinations included both Gram-negative and Gram-positive species, the usually expected higher resistance of Gram-negative bacteria to disinfection was not observed here [51,52]. Disinfectant susceptibility depends on the specific antimicrobial agent and experimental conditions, with biofilm structure and interspecies interactions potentially playing a greater role [37,53]. For combinations 1 to 3, bacterial proportions after disinfection with products A and C resembled those of the control, with *S. nepalensis* dominating combinations 1 and 2 and *C. freundii* in combination 3, indicating their lower sensitivity. *P. aeruginosa* decreased across treatments, while a slight increase in *Microbacterium* was observed after product C on PC coupons. This persistence, likely driven by strong biofilms formed by the dominant species (even after disinfection), such as *S. nepalensis* and *C. freundii*, highlights a key limitation of the treatments.

### 4.2 *In situ* monitoring of cleaning and disinfection protocols for livestock drinking water systems

The same five C&D protocols (A-E), additionally with chlorine gas treatment (F), performed during the downtime of four consecutive production rounds in a broiler house, were compared by collecting biofilm and drinking water samples after C&D and at two and five weeks of production. Control water samples showed a higher microbial load after C&D and after two weeks of production compared to the influent drinking water, indicating contamination within the water system. Using tap water as a source, products A (H_2_O_2_) and E (enzymes + PAA) improved microbial water quality only immediately after C&D, while additional reductions at two weeks were observed for products C (H_2_O_2_ + FA) and F (Cl_2_). Concerning biofilm samples, significantly lower loads than the control were observed only for products A and D (ClO_2_) at one time point (after C&D and after 5 weeks, respectively). In contrast, rainwater samples showed no consistent reductions compared to the controls. Results indicate that the performed C&D protocols had little to no impact on biofilm removal in DWS under practice conditions.

Although product A was less effective in the *in vitro* model for three of the four multispecies combinations, it performed better in practice, highlighting the additional importance of *in situ* validation. The *in vitro* models were used to represent simplified DWS, with selected strong biofilm-forming isolates grown under optimized conditions to produce biofilms within 48h. In contrast, the *in situ* biofilms in DWS are influenced by a wide range of uncontrolled and often unmeasured variables, including temperature fluctuations, microbial and chemical composition of the water, DWS material, nutrient availability, and the use of antibiotics and other supplements in the water [4,16,[54], [55], [56]]. An additional factor that may contribute to the observed differences is the distinct microbial community composition of the biofilms. Biofilms in DWS facilities are likely to comprise more diverse and complex microbial communities, with species interactions that may affect biofilm structure, resilience, and susceptibility to the products. Therefore, the simplified community composition of the *in vitro* models may not fully capture the interactions and ecological conditions present in native DWS biofilms, which could partly explain the difference in product effectiveness [32]. Furthermore, the sampling procedures may further affect measured outcomes. Biofilm can accumulate locally, which may or may not result in it being picked up by the cotton swabs. In addition, C&D protocols can detach biofilm flocs, which may or may not end up in the drinking water samples [57]. In addition, variations in the sampled surface area and swabbing pressure may have affected biofilm recovery, particularly given the water lines' curved geometry. Both *in vitro* and *in situ* approaches indicate that enzymatic treatment followed by peracetic acid treatment is effective, likely because enzymes disrupt the EPS matrix, thereby enhancing the subsequent disinfection step [[58], [59], [60]]. This method of C&D is not yet widely used in livestock barns, but it shows promise as an effective protocol.

Microbial water quality and biofilm levels in pipelines often return to pre-treatment conditions as the production cycle progresses. For example, one study reported no reduction in aerobic mesophilic counts in water samples collected before and after treatment with ClO_2_ in one poultry farm, or with peroxyacetic acid combined with ClO_2_ in another study [61]. Similarly, in a chlorine-treated poultry DWS, viable biofilm cells in waterlines rebounded to >4 log CFU/mL by the end of the first week [62]. Persistent biofilms, particularly on the inner surfaces of older DWS infrastructure, are also difficult to remove or eradicate [54,63,64]. To date, no widely accepted solution exists for preventing or removing biofilm formation in these systems; therefore, alternative or complementary control strategies could be implemented. Some examples include enzymatic treatments followed by disinfection, continuous dosing of water treatments [[65], [66], [67]], electrolyzed water [68,69], mechanical removal methods such as under high shear stress and ultrasonication [54,[70], [71], [72]] and targeted strategies against dominant biofilm-associated genera such as *Staphylococcus* and *Pseudomonas* [5,73,74].

## 5 Conclusion

Controlling and preventing persistent biofilms in livestock drinking water systems remain major challenges for maintaining high microbial water quality. Although various C&D strategies during production downtime are available, most are designed primarily to clean pipelines rather than target biofilms. In the study, two of the three hydrogen peroxide-based products performed poorly *in vitro* against multispecies biofilms containing the key synergistic species from pig nursery units, *C. freundii* and *S. nepalensis*. By contrast, three other disinfectants (hydrogen peroxide with PAA, chlorine dioxide, and enzymatic followed by PAA disinfection) eradicated all biofilm cells. However, these five disinfectants eradicated a multispecies combination from the broiler without the above-mentioned key species, highlighting the dependence of resistance on microbial composition. In the broiler pilot farm, where the same five disinfectants were tested alongside chlorine gas, the highest hydrogen peroxide dose reduced viable biofilm cells. Furthermore, disinfectants that were effective *in vitro* did not perform *in situ*, highlighting the strong influence of environmental factors and species interactions that are difficult to reproduce in laboratory settings. A combined protocol that used enzymatic biofilm degradation followed by PAA disinfection showed promising results in both cases, suggesting its potential for practical application. Taken together, these findings underscore the need for biofilm-oriented disinfection strategies, routine water-quality monitoring at both the source and the point-of-use, and systematic assessment of biofilm presence in water lines to enable timely and effective interventions.

## CRediT authorship contribution statement

**Ulric Van Rossum:** Writing – original draft, Methodology, Investigation, Formal analysis, Conceptualization. **Marc Heyndrickx:** Writing – review & editing, Supervision. **Niels Demaître:** Writing – review & editing. **Geertrui Rasschaert:** Writing – review & editing, Resources, Methodology, Funding acquisition. **Nico Boon:** Writing – review & editing, Supervision. **An Cools:** Writing – review & editing, Project administration, Funding acquisition. **Koen De Reu:** Writing – review & editing, Supervision, Methodology, Funding acquisition.

## Declaration of generative AI use

During the preparation of this work, the author used Microsoft Copilot and ChatGPT to improve language and readability, and assist with reference searches. After using this tool/service, the author reviewed and edited the content as needed and takes full responsibility for the content of the published article.

## Funding sources

This work was supported by the Flemish Agency for Innovation & Entrepreneurship - Agriculture & Agri-food (VLAIO-LA) [HBC.2021.1060] under the Blue Deal measures.

## Declaration of competing interest

The authors declare that they have no known competing financial interests or personal relationships that could have appeared to influence the work reported in this paper.

## Data Availability

All data generated or analyzed during this study are included in this article (and its Supplementary Table A1).
