# Supplementary material for: Laboratory and pilot-farm evaluation of cleaning and disinfection protocols for biofilms in livestock drinking water systems
Source: Biofilm. 2026 Sep 11;12:100397. doi: 10.1016/j.bioflm.2026.100397 (PMC13595119; doi:10.1016/j.bioflm.2026.100397)
Supplement: Supplementary file 1 [file mmc1.docx]

# 7. Supplementary Materials

**Table A1** Number of samples (n) taken at the pilot farm.

|  |  | Biofilm samples (n) | | | Drinking water samples (n) | | |
| --- | --- | --- | --- | --- | --- | --- | --- |
| Moment of sampling (weeks) | | 0 | 2 | 5 | 0 | 2 | 5 |
| Source | Tap | n.a. | n.a. | n.a. | 7 | 7 | 8 |
|  | Rain | n.a. | n.a. | n.a. | 4 | 3 | 4 |
| Control | Tap | 36 | 22 | 25 | 12 | 9 | 12 |
|  | Rain | 14 | 10 | 11 | 4 | 3 | 4 |
| A. | Tap | 14 | 10 | 11 | 4 | 3 | 4 |
|  | Rain | 14 | 10 | 11 | 4 | 3 | 4 |
| B. | Tap | 14 | 10 | 11 | 4 | 3 | 4 |
|  | Rain | 14 | 10 | 11 | 4 | 3 | 4 |
| C. | Tap | 22 | 12 | 14 | 8 | 6 | 8 |
| D. | Tap | 14 | 10 | 11 | 4 | 3 | 4 |
|  | Rain | 14 | 10 | 11 | 4 | 3 | 4 |
| E. | Tap | 22 | 12 | 14 | 8 | 6 | 8 |
| F. | Tap | 22 | 12 | 14 | 8 | 6 | 8 |

n.a. = not applicable

**Table A2** P-values for differences in biofilm cell viability between stainless steel (SS) and polycarbonate (PC) coupons over time, determined by two-way ANOVA followed by Šídák’s multiple-comparisons test.

| Source of variation | Combination | | | |
| --- | --- | --- | --- | --- |
|  | 1 | 2 | 3 | 4 |
| Time | <0.0001 | <0.0001 | <0.0001 | <0.0001 |
| SS vs PC | 0.0006 | 0.0121 | 0.0377 | 0.2196 |
| Time point SS vs PC |  |  |  |  |
| 0h | 0.9679 | 0.7761 | 0.4400 | 0.9647 |
| 4h | 0.1309 | 0.1722 | 0.1510 | 0.2147 |
| 7h | 0.2202 | 0.9802 | 0.9312 | 0.9398 |
| 24h | 0.0522 | 0.7597 | 0.0650 | 0.5859 |
| 28h | 0.0015 | 0.5791 | 0.7366 | >0.9999 |
| 31h | >0.9999 | 0.5822 | 0.9753 | 0.9260 |
| 48h | 0.7667 | 0.1392 | >0.9999 | >0.9999 |

**Table A3** P-values for differences in biofilm cell viability between the different multispecies combinations over time, determined by two-way ANOVA followed by Šídák’s multiple-comparisons test.

| Sampling time point | Combination comparison | | | | | |
| --- | --- | --- | --- | --- | --- | --- |
|  | 1 vs 2 | 1 vs 3 | 1 vs 4 | 2 vs 3 | 2 vs 4 | 3 vs 4 |
| Stainless steel |  |  |  |  |  |  |
| 0h | 0.9728 | >0.9999 | 0.9996 | 0.5623 | 0.3000 | >0.9999 |
| 4h | >0.9999 | 0.0158 | 0.9824 | 0.0097 | 0.9977 | 0.0078 |
| 7h | 0.9996 | 0.0206 | >0.9999 | 0.0100 | 0.9897 | 0.0065 |
| 24h | >0.9999 | 0.4002 | >0.9999 | 0.4667 | >0.9999 | 0.5430 |
| 28h | 0.9880 | 0.0124 | 0.5129 | 0.0732 | 0.7722 | 0.0849 |
| 31h | 0.1063 | 0.2782 | 0.0101 | 0.9019 | 0.0479 | 0.0682 |
| 48h | 0.305 | >0.9999 | 0.1054 | 0.2859 | 0.5120 | 0.0946 |
| Polycarbonate |  |  |  |  |  |  |
| 0h | 0.8719 | 0.7577 | 0.2431 | 0.4164 | 0.9989 | 0.1929 |
| 4h | 0.9728 | 0.0122 | 0.6714 | 0.4691 | 0.6124 | 0.0011 |
| 7h | 0.6045 | 0.3170 | 0.2503 | 0.0182 | 0.9926 | 0.0004 |
| 24h | 0.6873 | 0.9958 | 0.0528 | 0.8055 | >0.9999 | 0.1130 |
| 28h | 0.6263 | >0.9999 | 0.0062 | 0.7519 | 0.1233 | 0.0062 |
| 31h | 0.4074 | 0.6686 | 0.0625 | 0.9612 | 0.0223 | 0.0216 |
| 48h | 0.0091 | 0.3182 | 0.0001 | 0.6521 | 0.0018 | 0.0095 |
